# Tools for Diagnosing and Managing Sport-Related Concussion in UK Primary Care: A Scoping Review

**DOI:** 10.3390/sports13070201

**Published:** 2025-06-23

**Authors:** Sachin Bhandari, Soo Yit Gustin Mak, Neil Heron, John Rogers

**Affiliations:** 1 Salford Royal NHS Foundation Trust, Northern Care Alliance, Stott Lane, Salford M6 8HD, UK; 2 Barlow Medical Centre, 828 Wimslow Road, Manchester M20 2RN, UK; 3 Centre for Public Health, Queen’s University Belfast, Belfast BT12 6BA, UK; n.heron@qub.ac.uk; 4 School of Medicine, Keele University, Staffordshire ST5 5BG, UK; 5 OrthTeam Centre, 168 Barlow Moor Road, Manchester M20 2ZA, UK; 6 Department of Health Professions, Faculty of Health and Education, Manchester Metropolitan University, Manchester M15 6BX, UK

**Keywords:** sports-related concussion, primary care, general practice, diagnosis, management

## Abstract

Background: The UK Department for Digital, Culture, Media, and Sport (DCMS) grassroots concussion guidance, May 2023, advised that all community-based sport-related concussions (SRCs) be diagnosed by a healthcare practitioner. This may require that general practitioners (GPs) diagnose and manage SRCs. Diagnosing SRCs in primary care settings in the United Kingdom (UK) presents significant challenges, primarily due to the lack of validated tools specifically designed for general practitioners (GPs). This scoping review aims to identify diagnostic and management tools for SRCs in grassroots sports and primary care settings. Aims: To identify tools that can be used by GPs to diagnose and manage concussions in primary care, both adult and paediatric populations. Design and Methods: A scoping review was conducted in accordance with the Preferred Reporting Items for Systematic Reviews and Meta-Analyses extension for Scoping Reviews (PRISMA-ScRs). Five databases (MEDLINE, EMBASE, CINAHL, Cochrane Library, Google Scholar) were searched from 1946 to April 2025. Search terms included “concussion”, “primary care”, and “diagnosis”. Studies that discussed SRCs in community or primary care settings were included. Those that exclusively discussed secondary care and elite sports were excluded, as well as non-English studies. Two reviewers independently screened titles, abstracts, and full texts, with a third resolving any disagreements. Data were extracted into Microsoft Excel. Studies were assessed for quality using the Joanna Briggs critical appraisal tools and AGREE II checklist. Results: Of 727 studies, 12 met the inclusion criteria. Identified tools included Sport Concussion Assessment Tool 6 (SCAT6, 10–15 min, adolescent/adults), Sport Concussion Office Assessment Tool 6 (SCOAT6, 45–60 min, multidisciplinary), the Buffalo Concussion Physical Examination (BCPE, 5–6 min, adolescent-focused), and the Brain Injury Screening Tool (BIST, 6 min, ages 8+). As part of BCPE, a separate Telehealth version was developed for remote consultations. SCAT6 and SCOAT6 are designed for healthcare professionals, including GPs, but require additional training and time beyond typical UK consultation lengths (9.2 min). BIST and BCPE show promise but require UK validation. Conclusions: SCAT6, SCOAT6, BIST, and BCPE could enhance SRC care, but their feasibility in UK primary care requires adaptation (e.g., integration with GP IT systems and alignment with NICE guidelines). Further research is required to validate these tools and assess additional training needs.

## 1. Introduction

SRC, defined as a “traumatic brain injury caused by a direct blow to the head, neck or body resulting in an impulsive force being transmitted to the brain” [1], is a growing concern in grassroots sports. Characterised by non-specific symptoms that evolve over days, SRC lacks a definitive diagnostic test, posing challenges for timely and accurate assessment [1]. In the UK, the 2023 DCMS grassroots concussion guidance mandates that all community SRC cases be diagnosed by healthcare practitioners [2], increasingly directing patients to GPs via self-referral, 111 triage, or emergency departments. This policy is a response to a 2021 DCMS inquiry highlighting the absence of community-specific guidelines, variable clinician knowledge, and inadequate data collection, contributing to mismanagement, such as inappropriate return-to-play decisions [3].

GPs, often the initial point of contact for health issues [4], encounter significant barriers in delivering effective care. Rising workloads, a 19% increase from 2015 to 2023 [5], and brief consultation times (averaging 9.2 min in the UK) [6] limit patient-to-doctor time and primary care consultations. A survey of Irish GPs revealed that while most encounter SRC cases (1–5 annually), only 10% have had formal training in concussion diagnosis and management. In concussion diagnosis and management, indeed, three-quarters of these GPs provided return-to-play (RTP) guidance despite only 10% reporting adequate knowledge of RTP protocols [4]. Worryingly, one-fifth advised immediate return to play when patients were asymptomatic, without using any RTP protocol [4]. This indicates the need for accessible tools and education, particularly for paediatric and adult presentations with differing symptom profiles [7]. Tools like Sport Concussion Assessment Tool 6 (SCAT6, adolescents/adults, 10–15 min) and Sport Concussion Office Assessment Tool 6 (SCOAT6, subacute, 45–60 min), designed for healthcare professionals, including GPs, are time intensive and training dependent, reducing practicality in routine practice [8,9].

This scoping review seeks to identify diagnostic and management tools for SRCs suitable for use by GPs in primary care, for both adult and paediatric populations. By addressing the gap in UK-validated tools, it aims to support standardised care, enhance data collection, and improve patient outcomes in a resource-constrained environment.

## 2. Methods

This scoping review adhered to the Arksey and O’Malley framework [10,11] and PRISMA-ScR guidelines [12] (see Supplementary S1), although no formal protocol was registered.

Research Question:

What tools are used internationally for the diagnosis and management of SRCs in grassroots sports, primary care, and the community for adult and paediatric populations?

Search Strategy:

Five databases (MEDLINE, EMBASE, CINAHL, Cochrane Library, Google Scholar) were searched from 1946 to April 2025, with assistance from a medical librarian specialising in medical research to ensure a comprehensive search was conducted.

Key terms included (concussion OR head injury OR mild traumatic head injury) AND (grassroot OR community OR primary care OR non elite OR clinic OR accident and emergency OR emergency department) AND (assessment OR diagnosis OR monitoring)

Google Scholar searches used broader terms (e.g., concussion community assessment tool).

Inclusion: Studies discussing SRC diagnosis/management, conducted in primary care/grassroots settings, including adult/paediatric populations, published in English

Exclusion: Studies exclusively in secondary care/elite sports, non-original research (e.g., reviews, editorials), not published in English, or inaccessible full texts.

Data Extraction and Quality Assessment:

Data were extracted into Microsoft Excel, including author, year, time, study design, population, tools and target population, and management recommendations (see Table 1). Study quality was assessed using the Joanna Briggs critical appraisal tools and AGREE II checklists [13,14]. Bias was minimised through independent review and predefined criteria.

## 3. Results: Evaluation of Guidelines and Tools

**Figure 1:**
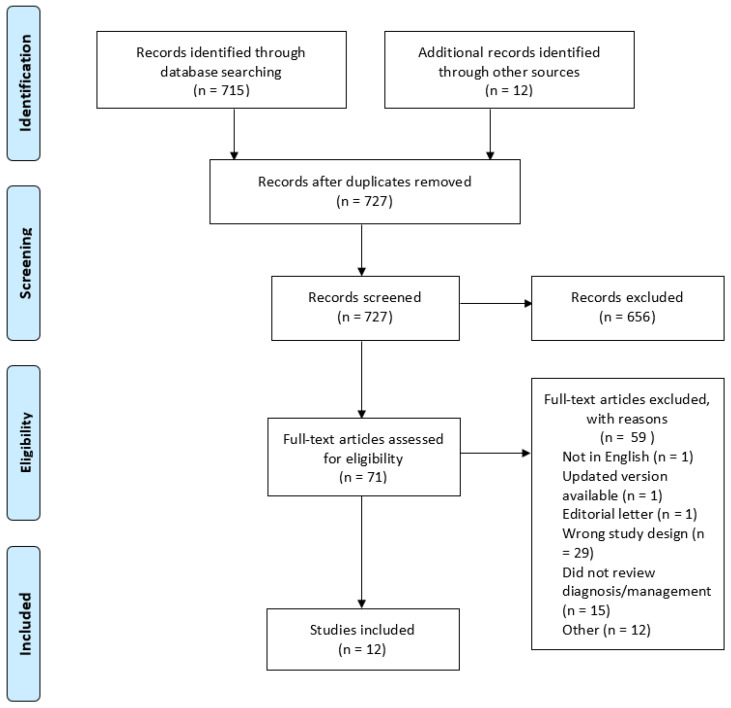
Prisma flowchart for diagnosis and management of sports-related concussion for general practitioners—a scoping review.

From 727 identified studies, two authors (SB and SM) independently screened titles and abstracts. Disagreements were resolved through discussion or by a third reviewer (NH). A full-text review was conducted on 71 articles. Reasons for exclusion included secondary care focus (1 article), inaccessible full texts (12 articles), not published in English (1 article), updated version published (1 article), editorial letter (1 article), or protocol (1 article). Additional exclusion reasons included a review or discussion paper (27 articles) or no review of diagnosis or management (15 articles). A total of 12 articles were included in this scoping review. A PRISMA flowchart (see Figure 1) summarises the process [28].

The scoping review identified four additional tools and one guideline relevant to the diagnosis and management of SRCs in primary care. An evaluation of these tools was conducted to gain insight into their available psychometric properties, validation status, alignment with the NICE head injury guidelines [29], and feasibility within UK general practice. Notably, none of the tools were specifically validated for use in UK primary care, thus highlighting a critical gap.

Living Concussion Guidelines: A Canadian resource regularly maintained by the Ontario Ministry of Health offers evidence-based recommendations for managing all concussion subtypes, including SRCs [15]. There are two separate guidelines available: one for the paediatric population (5–18 years) and a second for the adult population [15,16]. The guidelines assist healthcare professionals, including primary care clinicians, in diagnosing all causes of concussion [15,16]. Embedded within the Living Concussion guidelines for adults (aged 18 years and older) is the Acute Concussion Evaluation (ACE) form (see Supplementary S2) [30]. This ACE form explores symptoms via a checklist as well as risk factors for prolonged recovery to help with diagnosis (Supplementary S2), with any score more than 0 indicating a positive symptom history [30]. Although a user-friendly, practical resource, there are limited psychometric data giving insight into the specific sensitivity and specificity. There are no studies validating the applicability of the guidelines in UK primary care. The ACE tool incorporates many of the NICE head injury red flag symptoms [29,30]; however, this section would require slight adaptation to ensure incorporation of all NICE red flag criteria.

BCPE: A physical examination, supplemented with the Buffalo Concussion Treadmill Test (BCTT) if additional testing is required, is utilised for adolescents (aged 13–19 years) to assess concussion [19]. Studies have demonstrated, by assessing oculomotor and vestibular signs, components included in the BCPE, the ability to distinguish between suspected concussion and healthy controls within the first week following injury [19,21,31]. Donner et al. propose that a VVE should be included as part of the SRC assessment due to its reliability and sensitivity in the paediatric population [18]. Furthermore, it was found that the BCPE had 85% predictive accuracy in identifying children at low, medium, or high risk for delayed recovery [32]. The full brief physical examination is described by Haider et al., and the brief Buffalo Concussion Physical Exam Assessment (BCPE) form is freely available (Supplementary S3) [19]. After measuring orthostatic vital signs, the physical examination should take no more than 5 min to perform [19]. It is important to caveat that these studies were not conducted in primary care, and there are no supporting adult data. From a feasibility standpoint, the tool suits the time constraints of UK primary care consultations; however, it is yet to be seen whether it could be utilised across heterogenous cohorts, how much education clinicians would require on its use, supplemental equipment costs, and whether it could be integrated within primary care IT systems. Lack of an incorporated symptom tool to screen for red flags could pose a challenge.

Tele-BCPE: A Telehealth version of the BCPE (Supplementary S4) was developed during the COVID-19 pandemic when virtual consultations were implemented to reduce the risk of COVID virus transmission [22]. The examination took 15 min to administer [22]. The reliability and validity of this examination are not known.

BIST: A 6 min symptom-based screening tool, created by the Auckland University of Technology (AUT) Brain Injury Network for assessing anyone over the age of 8 years old to triage high-risk cases, diagnoses and monitors the recovery of those who have sustained a suspected concussion [33]. It is not specific to SRCs. The recommendation is to use this tool with additional questions or examinations, including balance or visual tests [33]. The BIST comprises 12 clinical indicator questions and a symptom checklist that spans physical, emotional, cognitive, and vestibular domains. These domains have been validated for concurrent validity with tools such as the Rivermead Post-Concussion Symptoms Questionnaire [27]. No studies have reported specific sensitivity or specificity data. Compared to the risk factors that prompt community healthcare workers to refer to emergency departments as recommended by NICE guidelines for head injury assessment, the BIST does not include amnesia for events before and after injury, a persistent headache since the injury, current drug or alcohol intoxication, or previous brain surgery [29,33]. These areas would need to be added to BIST-2 for it to be used routinely within the UK. From a feasibility standpoint, the examination is brief but effectively triages patients, it is user-friendly and would require minimal training, and it would likely be straightforward to implement into UK primary care IT systems.

Digital Tools: HeadCheck, a smartphone-based application developed in Australia, consists of two components: Recognition and Recovery [17]. It is used for children aged 5–18 and aimed at parents/community sport organisers. Validated tools such as SCAT5 and childSCAT5 are incorporated [17]. The app is not freely available in the UK. As a result, no further data or information could be obtained. Similarly, the New Zealand Rugby Concussion Assessment (NZRCA) is an application designed to record normative cognitive data to help clinicians delineate a deviation from baseline and place the individual on a standardised concussion management pathway [23]. This has yet to be validated. Both these tools are in the early stages of development, at the grassroots level, and require further research.

## 4. Discussion: Guiding Clinical Practice

The 2023 DCMS guidance has significantly raised awareness of SRCs at the grassroots and community levels [2]. Given that GPs are already grappling with substantial workloads, it is imperative to adopt a proactive approach to pre-emptively prepare for the anticipated surge in SRC presentations. It is evident that there is a pressing need for a comprehensive tool that can seamlessly integrate within the primary care network and be consistently completed within the timeframe (approximately 10 min) of an average primary care consultation. This tool should effectively aid in diagnosing, triaging, and managing SRC cases. This scoping review has identified a number of tools and evaluated them based on specific criteria. It is important to consider the variables faced in primary care. Patients may present at different times following a concussion, recover at different rates, and different tools might be better suited to acute or delayed presentations.

### 4.1. 0–72 h Post-Injury: Optimising Acute Diagnosis

The initial 72 h period following a suspected SRC is a critical window for identifying post-concussive symptoms and NICE red flags [29]. SCAT6 is sensitive for detecting symptoms and balance deficits but cannot be performed correctly in less than 10 to 15 min [8], exceeding typical primary care consultation times of 10 min [6]. Prioritisation of key components, for example, symptom checklist and red-flag assessments, is required. However, this complicates matters, leaving assessment down to the clinician’s discretion, risking inconsistent practices. The BIST is a quick and effective way to triage high-risk cases but omits NICE red flags such as focal neurological symptoms, thus limiting its standalone use [29,33]. Slightly adapting BIST to include NICE-specific red flag criteria and pairing it with condensed elements of the SCAT6 could offer a standardised approach for clinicians to identify high-risk cases and streamline acute assessments in time-constrained settings. Additionally, embedding flowcharts in primary care IT systems could further aid decision-making and reduce diagnostic variability. Further research is required to confirm this.

### 4.2. 72 h to 2 Weeks: Monitoring Recovery

From 72 h to 2 weeks, an effective tool must provide quantitative data to track symptom progression and guide clinicians to optimise recovery, while integrating with primary care workflows. SCOAT6 offers a comprehensive approach in serial monitoring [1], although it takes 45–60 min, making it impractical for a single practitioner [9]. Delegating components to other members of the team could enhance feasibility, akin to diagnostic workflows. However, UK primary care faces a shortage of appropriately trained healthcare professionals, particularly GPs, and this is therefore not practical currently. BCPE offers a quick approach, although only validated on paediatric populations, leveraging routine observations to detect autonomic dysfunction [19]. It promises brevity, but that comes at the cost of potential training requirements in tandem with gait and eye-tracking examinations for the untrained GP. The Living Concussion Guidelines offer simple and concise evidence-backed management advice, which can supplement tools and knowledge gaps [15]. Digitising SCOAT6 checklists or BCPE orthostatic observations into patient-facing apps could enable pre-consultation tracking, reducing workload and improving information availability, but is reliant on patient engagement with recovery.

### 4.3. 2–4 Weeks: Guiding Return-to-Play (RTP)

Weeks 2 to 4 represent a key transition point in care. Tools must aid clinicians in assessing symptom resolution to ensure safe return-to-play (RTP) and, indeed, safe return to life, education and work. SCAT6 and SCOAT6 provide structured protocols assessing symptoms, cognition, and balance [1,8,9], but time constraints persist. The Living Concussion Guidelines offer evidence-based RTP strategies for adults and children [15,16]. Only 43% of clinicians use standardised tools currently to guide management, citing complexity and training gaps. This is a barrier that must be overcome for effective implementation [20]. Educational interventions can promote clinician confidence in using SCAT6 or SCOAT6, but systemic solutions remain fundamental [26]. Embedding RTP algorithms into primary care IT systems, such as EMIS templates, makes information easily accessible and removes resistance for clinicians to check clearance criteria. Not only would this promote compliance with NICE guidance, but it would also ensure medico-legal standards are adhered to and avoid unnecessary premature RTP risks.

### 4.4. Managing Prolonged Symptoms

For those suffering from prolonged symptoms, tools must predict recovery trajectories and guide appropriate referrals in accordance with the guidance provided by the DCMS, that individuals exhibiting symptoms that persist beyond a 21-day period should be referred to a specialist healthcare professional. As part of the BCPE, a Risk for Delayed Recovery (RDR) score can be calculated around 2 weeks post-injury to identify children at risk of persistent post-concussive symptoms (PPCS), facilitating early specialist referral [19,32]. No equivalent adult-specific tools exist at present, leaving GPs reliant on clinical judgement for recognising PPCS. Although BIST monitors symptoms, it lacks validated sensitivity for detecting PPCS, thereby limiting its utility [27]. The Living Concussion Guidelines provide management strategies for dealing with such symptoms, but are limited in their predictive capabilities [15]. Future innovation and development of an adult-focused RDR equivalent is vital. Adapting the BCPE’s framework could offer a potential route. As improvements are made, linking predictive tools with referral pathways could mitigate delays. Until such tools emerge, a targeted PPCS screening question could be combined with current tools to identify referral needs. It would be necessary for GPs to receive training and be equipped with follow-up specialist clinics to address these referrals effectively.

## 5. Barriers to Tool Adoption

Three of the studies from the literature search utilised cross-sectional surveys to ascertain the current knowledge base of SRCs amongst clinicians and how this translated into clinical practice. Across the studies, utilisation of the SCATs remained limited, largely due to perceived complexity and training gaps [20,24,25]. Stoller et al. reported that 50% of family physicians avoided SCATs, even when aware of them [24]. Training alone is insufficient if tools feel misaligned with workflows. Adapting the aforementioned tools into user-friendly algorithms within primary care workflows, utilising IT systems, such as EMIS, could act as the cornerstone in identifying red flags, standardising concussion practice, ensuring proper documentation, and aiding continuity of care. Patient-facing apps such as HeadCheck could enable symptom tracking pre-consultation, potentially reducing workloads. Recently, a 2-year government-backed trial has seen the development of a similar app in the UK called SportSmart [34], showing the promise that suitable infrastructure could be available in the near future. These solutions could leverage and build on existing infrastructure via a build-test-scale approach to be implemented across the primary care network within the UK.

## 6. Strengths, Limitations, and Future Directions

This study comprehensively charts international practices of SRC diagnosis and management for primary care, supported by input from clinicians in primary care, secondary care, and sports medicine. However, its reliance on 12 studies reflects a global paucity of primary care-focused SRCs research. A total of 12 studies were inaccessible, possibly due to the age of the research, despite using an academic librarian backed by a research institution to conduct the search. In addition, one paper was not available in English. It is plausible that the inability to review these studies may have omitted crucial insights. Future research should prioritise validating SCAT6, SCOAT6, BCPE, and BIST within primary care consultations, particularly within the time constraints faced by primary care practitioners. Studies should assess the sensitivity of these tools in detecting SRCs and general usability. Pilot studies integrating adapted tools with primary care IT systems could confirm feasibility while addressing training and workflow barriers. Cost–benefit analysis should also be conducted to ascertain whether tool adoption and application creation would be economically feasible.

## 7. Conclusions

It is anticipated that SRC presentations to primary care will increase following the release of the 2023 DCMS grassroots concussion guidance in the UK. It is vital that GPs are adequately equipped with effective and efficient tools that align with their workflows. SCOAT6 and SCAT6 are effective, but cannot be carried out within the typical 10 min consultation time of UK general practice. BIST and BCPE offer quicker alternatives but lack UK validation and alignment with NICE head injury advice. The Living Concussion Guidelines provide practical management for recovery and persistent symptoms. A UK-adapted tool, incorporating the BIST’s symptom triage, BCPE physical exam, and NICE red flag criteria, should be developed as a template on primary care IT systems for seamless integration. Educational and training initiatives are pivotal to address knowledge gaps and improve clinician confidence in implementing tools effectively. Further research should prioritise pilot studies to assess tool accuracy, feasibility, and patient outcomes in UK primary care. This will help establish standardised care practices and facilitate robust data collection on concussion diagnosis and management, which can then be used to create feedback loops and guide future iterations in practice for long-term concussion management.

**Key Findings:**

- No UK-validated SRCs tools exist specifically for primary care, despite DCMS guidance on concussion management within the community.
- SCAT6 and SCOAT6 are suitable for GPs but time-intensive; BIST and BCPE are quicker but not validated in the UK setting.
- Development of standardised concussion assessment tools for use in UK primary care could improve concussion diagnosis and management, with improved patient outcomes.

## Figures and Tables

**Table 1:** Summary of studies included.

Author (Year)	Tool/Strategy	Study Design	Population/Target	Diagnostic Criteria	Management Recommendations	Quality Assessment
Living Concussion Guidelines (2019) [15,16]	Living Concussion Guidelines (ACE tool)	Guidelines	Adults (18+), paediatrics (5–18); primary care providers	ACE symptom checklist; no minimum score	Symptom management, RTP advice	AGREE II: adults (7), paediatrics (6)
Clarke, C., et al. (2020) [17]	HeadCheck App	Digital health application	Paediatrics (5–18); community members	Using child SCAT-5 as reference	RTP and school advice	JBI critical appraisal checklist for qualitative research: high score
Donner, J. R., et al. (2023) [18]	Visio-vestibular examination (VVE)	Retrospective chart review	Paediatric (5–18); Emergency department, Primary care	VVE (smooth pursuit, saccades, gaze stability)	Anticipatory guidance: 67.9% brain rest and 72.8% recommended physical rest	JBI critical appraisal checklist for cohort study used to assess quality: high score but some bias as examination template used in primary care setting
Haider et al. (2020) [19]	BCPE	Descriptive examination; refer to Leddy et al. for derivation of this examination	Adolescents (13–19); outpatient clinics	Orthostatic examination followed by 5 min. Examination of (cranial nerves, oculomotor, etc.)	None	N/A (descriptive)
Lebrun, C. M., et al. (2013) [20]	SRC knowledge survey	Cross-sectional survey	Family physicians (US/CAN); primary care	SCAT 1 and SCAT 2. Clinical exam and balance testing	RTP guidance/education needs	Low response rate, 2 separate sites used to account for differences in training programmes. Prone to volunteer and recall bias
Leddy et al. (2018) [21]	Derivation of BCPE	Prospective cohort study	Adolescents (13–19); outpatient clinics	Recommendations for BCPE. Carried out as part of a randomised control trial of provocative exercise testing (Buffalo Concussion Treadmill Test)	None	JB critical appraisal checklist for Cohort study used to assess quality: high score
McPherson, J. I., et al. (2022) [22]	Tele-BCPE	Descriptive tele-examination	Adolescents (13–19); outpatient clinics	Modified BCPE screening for orthostatic intolerance and tests cranial nerves, oculomotor, vestibular, and cervical systems	None	No validity or reliability studies conducted. Derived from Buffalo Concussion Physical Examination. (See Leddy et al. and Haider et al.)
Salmon, D. M., et al. (2022) [23]	NZRCA App	Prospective cross-sectional	High school rugby players; primary care	A subset of neurocognitive assessment components from SCAT5, ChildSCAT5, and SCAT3 for the development of NZRCA app for baseline testing	RTP guidance and community concussion management pathway	Robust sample size. Accounts for individual differences. Limitations: baseline tests were conducted, smaller sample size for females and the schools selected for the initial pilot
Stoller, J., et al. (2014) [24]	SRC management survey	Cross-sectional survey	Emergency care, primary care, and paediatric care settings	SCAT (rarely used), clinical judgement, and diagnostic imaging	Address knowledge transfer gaps	Survey sent to 10 methodologists and 10 typical participants for content validation. An iterative process ensured question relevance and clarity. Statistics tests checked for statistically significant differences between groups
Stuart, C., et al. (2022) [25]	SRC knowledge survey	Cross-sectional survey	GPs/urgent care (New Zealand); primary Care	SCAT (43% use)	Education and IT-integrated tools	Members of the workforce may have chosen not to participate, lacking representation in the results. Clinicians with an interest in sport or SRCs are more likely to participate, potentially introducing biases
Taylor, A. M., et al. (2018) [26]	Concussion education	Qualitative	Paediatric primary care	Consensus-based guidelines	RTP and School	References best practice management guidelines from previous consensus statements
A Theadom et al. (2021) [27]	BIST	Tool validity testing and survey	Ages 8+; primary and secondary care	6 min symptom triage followed by risk stratification	Healthcare pathway guide	High concurrent validity against River Mead Questionnaire and SCAT-5

## Data Availability

Not applicable.

## Supplementary Materials

The following supporting information can be downloaded at: https://www.mdpi.com/article/10.3390/sports13070201/s1, Supplementary S1: Preferred Reporting Items for Systematic reviews and Meta-Analyses extension for Scoping Reviews (PRISMA-ScR) Checklist; Supplementary S2: Acute Concussion Evaluation (ACE) Form; Supplementary S3: Buffalo Concussion Physical Exam Assessment; Supplementary S4: Components of In-person BCPE and Tele-BCPE.
