# Supplementary material for: Tools for Diagnosing and Managing Sport-Related Concussion in UK Primary Care: A Scoping Review
Source: Sports (Basel). 2025 Jun 23;13(7):201. doi: 10.3390/sports13070201 (PMC12298353; doi:10.3390/sports13070201)
Supplement: Supplementary file 1 [file sports-13-00201-s001.zip › Supplementary material.pdf]

Supplementary S2: Acute Concussion Evaluation (ACE) Form (19)

Acute Concussion Evaluation (ACE): Physician/Clinician Office Version

Acute Concussion Evaluation (ACE)
Physician/Clinician Office Version

Gerard Gioia, PhD[1] & Micky Collins, PhD[2]
[1]Children's National Medical Center
[2]University of Pittsburgh Medical Center

Patient Name:______________________
DOB: __________ Age:______________
Date:__________ ID/MR#____________

A. Injury Characteristics Date/Time of Injury____________ Reporter: __Patient __Parent __Spouse __Other________

1. Injury Description ________________________________

1a. Is there evidence of a forcible blow to the head (direct or indirect)? __Yes __No __Unknown
1b. Is there evidence of intracranial injury or skull fracture? __Yes __No __Unknown
1c. Location of Impact: __Frontal __Lft Temporal __Rt Temporal __Lft Parietal __Rt Parietal __Occipital __Neck __Indirect Force
2. Cause: __MVC __Pedestrian-MVC __Fall __Assault __Sports (*specify*)____________ Other____________
3. Amnesia Before (Retrograde) Are there any events just BEFORE the injury that you/ person has no memory of (even brief)? _ Yes _No Duration
4. Amnesia After (Anterograde) Are there any events just AFTER the injury that you/ person has no memory of (even brief)? __ Yes __No Duration
5. Loss of Consciousness: Did you/ person lose consciousness? __ Yes __No Duration
6. EARLY SIGNS: __Appears dazed or stunned __Is confused about events __Answers questions slowly __Repeats Questions __Forgetful (recent info)
7. Seizures: Were seizures observed? No__ Yes___ Detail____________

B. Symptom Check List* Since the injury, has the person experienced any of these symptoms any more than usual today or in the past day?
Indicate presence of each symptom (0=No, 1=Yes). ****Lovell & Collins, 1998 JHTR***

PHYSICAL (10)		COGNITIVE (4)		SLEEP (4)	
Headache	0 1	Feeling mentally foggy	0 1	Drowsiness	0 1
Nausea	0 1	Feeling slowed down	0 1	Sleeping less than usual	0 1 N/A
Vomiting	0 1	Difficulty concentrating	0 1	Sleeping more than usual	0 1 N/A
Balance problems	0 1	Difficulty remembering	0 1	Trouble falling asleep	0 1 N/A
Dizziness	0 1	**COGNITIVE Total (0-4)** ____		**SLEEP Total (0-4)** ____	
Visual problems	0 1	**EMOTIONAL (4)**			
Fatigue	0 1	Irritability	0 1		
Sensitivity to light	0 1	Sadness	0 1		
Sensitivity to noise	0 1	More emotional	0 1		
Numbness/Tingling	0 1	Nervousness	0 1		
**PHYSICAL Total (0-10)** ____		**EMOTIONAL Total (0-4)** ____			
		**(Add Physical, Cognitive, Emotion, Sleep totals) Total Symptom Score (0-22)**	____		

Exertion: Do these symptoms worsen with:
Physical Activity __Yes __No __N/A
Cognitive Activity __Yes __No __N/A

Overall Rating: How different is the person acting compared to his/her usual self? (circle)
Normal 0 1 2 3 4 5 6 Very Different

C. Risk Factors for Protracted Recovery (*check all that apply*)

**Concussion History?** Y ___ N__	√	**Headache History?** Y ___ N__	√	**Developmental History**	√	**Psychiatric History**
Previous # 1 2 3 4 5 6+		Prior treatment for headache		Learning disabilities		Anxiety
Longest symptom duration Days__ Weeks__ Months__ Years__		History of migraine headache __ Personal __ Family________		Attention-Deficit/ Hyperactivity Disorder		Depression
						Sleep disorder
If multiple concussions, less force caused reinjury? Yes__ No__		____________		Other developmental disorder________		Other psychiatric disorder ________

List other comorbid medical disorders or medication usage (e.g., hypothyroid, seizures)____________

D. RED FLAGS for acute emergency management: Refer to the emergency department with sudden onset of any of the following:
* Headaches that worsen
* Seizures
* Focal neurologic signs
* Looks very drowsy/ can't be awakened
* Repeated vomiting
* Slurred speech
* Can't recognize people or places
* Increasing confusion or irritability
* Weakness or numbness in arms/legs
* Neck pain
* Unusual behavioral change
* Change in state of consciousness

E. Diagnosis (ICD): __Concussion w/o LOC 850.0 __Concussion w/ LOC 850.1 __Concussion (Unspecified) 850.9 __Other (854) ________
__No diagnosis

F. Follow-Up Action Plan **Complete *ACE Care Plan* and provide copy to patient/family.**
___ **No Follow-Up Needed**
___ **Physician/Clinician Office Monitoring**: Date of next follow-up ____________
___ **Referral:**
___ Neuropsychological Testing
___ Physician: Neurosurgery____ Neurology____ Sports Medicine____ Physiatrist____ Psychiatrist____ Other____________
___ Emergency Department

ACE Completed by:____________________

© Copyright G. Gioia & M. Collins, 2006

This form is part of the "Heads Up: Brain Injury in Your Practice" tool kit developed by the Centers for Disease Control and Prevention (CDC).

ACE Instructions

The ACE is intended to provide an evidence-based clinical protocol to conduct an initial evaluation and diagnosis of patients (both children and adults) with known or suspected MTBI. The research evidence documenting the importance of these components in the evaluation of an MTBI is provided in the reference list.

A. Injury Characteristics:

1. Obtain **description of the injury** – how injury occurred, type of force, location on the head or body (if force transmitted to head). Different biomechanics of injury may result in differential symptom patterns (e.g., occipital blow may result in visual changes, balance difficulties).
2. Indicate the **cause of injury**. Greater forces associated with the trauma are likely to result in more severe presentation of symptoms.

3/4. **Amnesia:** Amnesia is defined as the failure to form new memories. Determine whether amnesia has occurred and attempt to determine length of time of memory dysfunction – before (retrograde) and after (anterograde) injury. Even seconds to minutes of memory loss can be predictive of outcome. Recent research has indicated that amnesia may be up to 4-10 times more predictive of symptoms and cognitive deficits following concussion than is LOC (less than 1 minute).[1]

5. **Loss of consciousness (LOC)** – If occurs, determine length of LOC.
6. **Early signs.** If present, ask the individuals who know the patient (parent, spouse, friend, etc) about specific signs of the concussion that may have been observed. These signs are typically observed early after the injury.
7. Inquire whether **seizures** were observed or not.

B. Symptom Checklist: [2]

1. Ask patient (and/or parent, if child) to report presence of the four categories of symptoms since injury. It is important to assess all listed symptoms as different parts of the brain control different functions. One or all symptoms may be present depending upon mechanisms of injury.[3] Record "1" for Yes or "0" for No for their presence or absence, respectively.
2. For all symptoms, indicate presence of symptoms as experienced within the past 24 hours. Since symptoms can be present premorbidly/at baseline (e.g., inattention, headaches, sleep, sadness), it is important to assess change from their usual presentation.
3. **Scoring**: Sum total number of symptoms present per area, and sum all four areas into Total Symptom Score (score range 0-22). (Note: most sleep symptoms are only applicable after a night has passed since the injury. Drowsiness may be present on the day of injury.) If symptoms are new and present, there is no lower limit symptom score. Any score > 0 indicates positive symptom history.
4. **Exertion:** Inquire whether any symptoms worsen with physical (e.g., running, climbing stairs, bike riding) and/or cognitive (e.g., academic studies, multi-tasking at work, reading or other tasks requiring focused concentration) exertion. Clinicians should be aware that symptoms will typically worsen or re-emerge with exertion, indicating incomplete recovery. Over-exertion may protract recovery.
5. **Overall Rating**: Determine how different the person is acting from their usual self. Circle "0" (Normal) to "6" (Very Different).

C. Risk Factors for Protracted Recovery: Assess the following risk factors as possible complicating factors in the recovery process.

1. **Concussion history:** Assess the number and date(s) of prior concussions, the duration of symptoms for each injury, and whether less biomechanical force resulted in re-injury. Research indicates that cognitive and symptom effects of concussion may be cumulative, especially if there is minimal duration of time between injuries and less biomechanical force results in subsequent concussion (which may indicate incomplete recovery from initial trauma).[4-8]
2. **Headache history:** Assess personal and/or family history of diagnosis/treatment for headaches. Research indicates headache (migraine in particular) can result in protracted recovery from concussion.[8-11]
3. **Developmental history**: Assess history of learning disabilities, Attention-Deficit/Hyperactivity Disorder or other developmental disorders. Research indicates that there is the possibility of a longer period of recovery with these conditions.[12]
4. **Psychiatric history**: Assess for history of depression/mood disorder, anxiety, and/or sleep disorder.[13-16]

D. Red Flags: The patient should be carefully observed over the first 24-48 hours for these serious signs. Red flags are to be assessed as possible signs of deteriorating neurological functioning. Any positive report should prompt strong consideration of referral for emergency medical evaluation (e.g. CT Scan to rule out intracranial bleed or other structural pathology).[17]

E. Diagnosis: The following ICD diagnostic codes may be applicable.

850.0 (Concussion, with no loss of consciousness) – Positive injury description with evidence of forcible direct/ indirect blow to the head (A1a); plus evidence of active symptoms (B) of any type and number related to the trauma (Total Symptom Score >0); no evidence of LOC (A5), skull fracture or intracranial injury (A1b).

850.1 (Concussion, with brief loss of consciousness < 1 hour) – Positive injury description with evidence of forcible direct/ indirect blow to the head (A1a); plus evidence of active symptoms (B) of any type and number related to the trauma (Total Symptom Score >0); positive evidence of LOC (A5), skull fracture or intracranial injury (A1b).

850.9 (Concussion, unspecified) – Positive injury description with evidence of forcible direct/ indirect blow to the head (A1a); plus evidence of active symptoms (B) of any type and number related to the trauma (Total Symptom Score >0); unclear/unknown injury details; unclear evidence of LOC (A5), no skull fracture or intracranial injury.

Other Diagnoses – If the patient presents with a positive injury description and associated symptoms, but additional evidence of intracranial injury (A 1b) such as from neuroimaging, a moderate TBI and the diagnostic category of 854 (Intracranial injury) should be considered.

F. Follow-Up Action Plan: Develop a follow-up plan of action for symptomatic patients. The physician/clinician may decide to (1) monitor the patient in the office or (2) refer them to a specialist. Serial evaluation of the concussion is critical as symptoms may resolve, worsen, or ebb and flow depending upon many factors (e.g., cognitive/physical exertion, comorbidities). Referral to a specialist can be particularly valuable to help manage certain aspects of the patient's condition. (Physician/Clinician should also complete the ACE Care Plan included in this tool kit.)

1. **Physician/Clinician serial monitoring –** Particularly appropriate if number and severity of symptoms are steadily decreasing over time and/or fully resolve within 3-5 days. If steady reduction is not evident, referral to a specialist is warranted.
2. **Referral to a specialist –** Appropriate if symptom reduction is not evident in 3-5 days, or sooner if symptom profile is concerning in type/severity.
   - Neuropsychological Testing can provide valuable information to help assess a patient's brain function and impairment and assist with treatment planning, such as return to play decisions.
   - Physician Evaluation is particularly relevant for medical evaluation and management of concussion. It is also critical for evaluating and managing focal neurologic, sensory, vestibular, and motor concerns. It may be useful for medication management (e.g., headaches, sleep disturbance, depression) if post-concussive problems persist.

Supplementary S3: Buffalo Concussion Physical Exam Assessment (20)

Table 1. Brief Buffalo Concussion Physical Exam Assessment Form Date: ______________

Orthostatic Vital Signs		
	**Supine**	**Standing (after 1 minute)**
Blood Pressure (mmHg)		
Heart Rate (bpm)		
Symptoms[1]	☐ No<br>☐ Yes<br>If yes: Description?	☐ No<br>☐ Yes<br>If yes: Description?
Results	☐ Normal ☐ Abnormal	
Test results are deemed clinically significant if they include at least one of the following AND symptomatic: (1) systolic BP drop of ≥ 20mmHg or (2) diastolic BP drop of ≥ 10mmHg.		

Cranial Nerve Exam		
☐ **Performed** ☐ **Not performed**		
**Nerve**	**Test**	**Results**
I – Olfactory	With eyes closed, have the patient plug one nostril and smell distinctive scent (e.g. coffee grounds), repeat on other side.	☐ Normal ☐ Abnormal
V – Trigeminal	With eyes closed, touch the patient on the forehead, cheek and jaw while asking if the sensation is the same bilaterally.	☐ Normal ☐ Abnormal
VII – Facial	Ask the patient to smile, puff out the cheeks, wrinkle the forehead, and close the eyes tightly.	☐ Normal ☐ Abnormal
IX – Glossopharyngeal	Ask the patient to open the mouth and say "ahhh."	☐ Normal ☐ Abnormal
X – Vagus	Ask the patient to swallow.	☐ Normal ☐ Abnormal
XI – Accessory	Push down lightly on the patient's shoulders while the patient shrugs.	☐ Normal ☐ Abnormal
XII - Hypoglossal	Ask the patient to stick out the tongue.	☐ Normal ☐ Abnormal

Oculomotor/Ophthalmologic Evaluation		
**Test**	**Results**	**Symptoms**
Fundoscopy	☐ Performed<br>☐ Not performed	☐ Normal<br>Other:
Pupil Reactivity (CN II)	☐ Normal ☐ Abnormal	
Visual Tracking (CN III, IV, VI)	☐ Normal ☐ Abnormal	
Smooth Pursuits<br>(10 repetitions and then stop, horizontal and vertical)	☐ Normal<br>☐ Abnormal Horizontal<br>☐ Abnormal Vertical	☐ Nystagmus[2] (sign)<br>☐ Saccadic movement (sign)<br>☐ Dizziness/nausea (symptom)<br>☐ Worse headache (symptom)<br>☐ Other:
Repetitive Saccades[3]<br>(30 repetitions horizontal, 30 repetitions vertical)	☐ Normal<br>☐ Abnormal Horizontal<br>☐ Abnormal Vertical	☐ Nystagmus[2] (sign)<br>☐ Saccadic movement (sign)<br>☐ Dizziness/nausea (symptom)<br>☐ Worse headache (symptom)<br>☐ Other:
VOR (CN VIII)[3]<br>(10 repetitions horizontal, 10 repetitions vertical)	☐ Normal<br>☐ Abnormal Horizontal<br>☐ Abnormal Vertical	☐ Does not maintain fixation (sign)<br>☐ Dizziness/nausea (symptom)<br>☐ Worse headache (symptom)<br>☐ Other:

Near-Point Convergence and Accommodation[4]				
**Trial**	**Convergence (diplopia)**	**Convergence Recovery (single image)**	**Accommodation – Right (blurry)**	**Accommodation – Left (blurry)**
1	cm	cm	cm	cm
2	cm	cm	cm	cm
Best	cm	cm	cm	cm

Neck and Sub-Occipital Region Exam		
**Palpitation[6]**	**Signs and Symptoms**	**Location**
Spasm	☐ Normal ☐ Abnormal	
Tenderness	☐ Normal ☐ Abnormal	
**Cervical Range of Motion**	**Result**	
Flexion (50°)	☐ Normal ☐ Abnormal	
Extension (60°)	☐ Normal ☐ Abnormal	
Right Lateral Flexion (40–45°)	☐ Normal ☐ Abnormal	
Left Lateral Flexion (40–45°)	☐ Normal ☐ Abnormal	
Right Rotation (80°)	☐ Normal ☐ Abnormal	
Left Rotation (80°)	☐ Normal ☐ Abnormal	

Postural Control and Motor Coordination			
**Test**	**Description**	**Result**	**Signs/Symptoms**
Tandem Gait Eyes Open (forward and backward)	Patient walks 5 steps forward and backward heel to toe while looking straight ahead.	☐ Normal<br>☐ Abnormal	☐ Fall/unstable (sign)<br>☐ Over step (sign)<br>☐ Dizziness/nausea (symptom)<br>☐ Other:
Tandem Gait Eyes Closed (forward and backward)	Patient walks 5 steps forward and backward heel to toe with eyes closed.	☐ Normal<br>☐ Abnormal	☐ Fall/unstable (sign)<br>☐ Over step (sign)<br>☐ Dizziness/nausea (symptom)<br>☐ Other:
Tandem Stance	Patient stops walking and stands for 20 seconds in a heel to toe stance with hands on the hips and eyes closed.	☐ Normal<br>☐ Abnormal	☐ Fall/unstable (sign)<br>☐ Dizziness/nausea (symptom)<br>☐ Other:

[illegible]

Supplementary S4: Components of In-person BCPE and Tele-BCPE (25)

**In-Person BCPE**	**Tele-BCPE**
Pre-assessment:<br>- Clinic Paperwork<br>- Concussion Symptom Checklist<br>- Orthostatic Vital Signs	Pre-assessment:<br>- Clinic Paperwork<br>- Concussion Symptom Checklist
History	History
N/A	Orthostatic Intolerance Screen
Cervical Exam<br>- Manual Palpation<br>- Range of Motion	Cervical Exam<br>- Self-guided palpation<br>- Range of Motion<br>- Cervical Rotation Flexion Test<br>-
Head and Face Exam<br>- Craniofacial and Scalp Screen	Head and Face Exam<br>- Self-guided Palpation
Cranial Nerve Exam<br>- Assess CN I-XII	Cranial Nerve Exam<br>Assess CN I-VII, XI, XII
Oculomotor Exam<br>- Smooth Pursuits<br>- Repetitive Saccades<br>- Near-point Convergence	Oculomotor Exam<br>- Smooth Pursuits<br>- Repetitive Saccades<br>- Near-point Convergence
Vestibular/Balance Exam<br>- Vestibulo-ocular Reflex<br>- Tandem Gait, Forward and Backward, Eyes Open, Eyes Closed	Vestibular/Balance Exam<br>- Vestibulo-ocular Reflex<br>- Sharpened Romberg Test<br>- Single Legged Stance
Adjunct Testing<br>- Fundoscopy<br>- Exercise Tolerance Test	Adjunct Testing<br>- Finger to Nose Test
